# Consumption of Grapes Modulates Gene Expression, Reduces Non-Alcoholic Fatty Liver Disease, and Extends Longevity in Female C57BL/6J Mice Provided with a High-Fat Western-Pattern Diet

**DOI:** 10.3390/foods11131984

**Published:** 2022-07-05

**Authors:** Asim Dave, Eun-Jung Park, Avinash Kumar, Falguni Parande, Diren Beyoğlu, Jeffrey R. Idle, John M. Pezzuto

**Affiliations:** 1 Division of Pharmaceutical Sciences, Arnold & Marie Schwartz College of Pharmacy and Health Sciences, Long Island University, Brooklyn, NY 11201, USA; asimbdave@gmail.com (A.D.); eunjung.park@liu.edu (E.-J.P.); avinash.kumar@liu.edu (A.K.); falguniparande@gmail.com (F.P.); 2 Immunology Program, Memorial Sloan Kettering Cancer Center, New York, NY 10065, USA; 3 Artus Therapeutics, Harvard Life Lab, Allston, MA 02134, USA; 4 Arthur G. Zupko’s Institute of Systems Pharmacology and Pharmacogenomics, Arnold and Marie Schwartz College of Pharmacy and Health Sciences, Long Island University, Brooklyn, NY 11201, USA; diren.beyoglu@liu.edu (D.B.); jeff.idle@liu.edu (J.R.I.); 5 College of Pharmacy and Health Sciences, Western New England University, Springfield, MA 01119, USA

**Keywords:** grapes, metabolic diseases, C57BL/6J mice, high-fat diet, RNA sequencing, nutrigenomics

## Abstract

A key objective of this study was to explore the potential of dietary grape consumption to modulate adverse effects caused by a high-fat (western-pattern) diet. Female C57BL/6J mice were purchased at six-weeks-of-age and placed on a standard (semi-synthetic) diet (STD). At 11 weeks-of-age, the mice were continued on the STD or placed on the STD supplemented with 5% standardized grape powder (STD5GP), a high-fat diet (HFD), or an HFD supplemented with 5% standardized grape powder (HFD5GP). After being provided with the respective diets for 13 additional weeks, the mice were euthanized, and liver was collected for biomarker analysis, determination of genetic expression (RNA-Seq), and histopathological examination. All four dietary groups demonstrated unique genetic expression patterns. Using pathway analysis tools (GO, KEGG and Reactome), relative to the STD group, differentially expressed genes of the STD5GP group were significantly enriched in RNA, mitochondria, and protein translation related pathways, as well as drug metabolism, glutathione, detoxification, and oxidative stress associated pathways. The expression of *Gstp1* was confirmed to be upregulated by about five-fold (RT-qPCR), and, based on RNA-Seq data, the expression of additional genes associated with the reduction of oxidative stress and detoxification (*Gpx4* and *8*, *Gss*, *Gpx7*, *Sod1*) were enhanced by dietary grape supplementation. Cluster analysis of genetic expression patterns revealed the greatest divergence between the HFD5GP and HFD groups. In the HFD5GP group, relative to the HFD group, 14 genes responsible for the metabolism, transportation, hydrolysis, and sequestration of fatty acids were upregulated. Conversely, genes responsible for lipid content and cholesterol synthesis (*Plin4*, *Acaa1b*, *Slc27a1*) were downregulated. The two top classifications emerging as enriched in the HFD5GP group vs. the HFD group (KEGG pathway analysis) were Alzheimer’s disease and nonalcoholic fatty liver disease (NAFLD), both of which have been reported in the literature to bear a causal relationship. In the current study, nonalcoholic steatohepatitis was indicated by histological observations that revealed archetype markers of fatty liver induced by the HFD. The adverse response was diminished by grape intervention. In addition to these studies, life-long survival was assessed with C57BL/6J mice. C57BL/6J mice were received at four-weeks-of-age and placed on the STD. At 14-weeks-of-age, the mice were divided into two groups (100 per group) and provided with the HFD or the HFD5GP. Relative to the HFD group, the survival time of the HFD5GP group was enhanced (log-rank test, *p* = 0.036). The respective hazard ratios were 0.715 (HFD5GP) and 1.397 (HFD). Greater body weight positively correlated with longevity; the highest body weight of the HFD5GP group was attained later in life than the HFD group (*p* = 0.141). These results suggest the potential of dietary grapes to modulate hepatic gene expression, prevent oxidative damage, induce fatty acid metabolism, ameliorate NAFLD, and increase longevity when co-administered with a high-fat diet.

## 1. Introduction

Nutrition and its relationship to health, disease and longevity is a theme that has been pondered and investigated over the millennia. Hallmark epidemiological studies, such as those reported by Doll [1], Phillips [2], and Dunn [3], among others, have provided a conceptual framework for understanding disease etiology and, by extension, guiding principles for disease prevention. Despite strong evidence and common sense, eradication of threats to health is a slow and cumbersome process. The ongoing smoking of cigarettes, irrespective of known risk, is a prime example. Similarly, aspects of the human diet, in particular the western-pattern diet [4], presents chronic risk that could be avoided. Conversely, the eloquent quotation attributed to Hippocrates, “Let food be thy medicine, and let medicine be thy food”, is less of a polemic. Mainstream contemporary thinking often includes the concept of the “healthy diet” [5]. Invariably, “healthy diet” commentaries include high levels of fruits and vegetables (e.g., five servings per day), and whole grains, concomitant with low trans-fat, saturated fats, and refined carbohydrates [6]. Explicitly, the US Department of Agriculture economics research service recommends including at least two cups of fruit and 2.5 cups of vegetables in a daily diet [7].

There is broad scientific underpinning in support of such recommendations. Fruits and vegetables have a crucial role in maintaining homeostasis and regulating pathological conditions that can be related to genic and epigenetic modifications. For example, a growing body of evidence suggests that fruits and vegetables can influence epigenetics by increasing or silencing gene expression. Furthermore, the expression of antioxidant phase II metabolism can be increased by fruits and vegetables. Activation of Nrf2, encoded by Nuclear Factor Erythroid derivative like 2 (NFE2L2) [8], induces transcription of genes encoding phase II antioxidant enzymes such as glutathione *S*-transferase A2 (GSTA2), glutathione *S*-transferase mu 1 (GSTM1), NAD(P)H: quinone oxidoreductase I (NQO1), glutamate cysteine ligase regulatory subunit (GCLM), glutamate cysteine ligase catalytic subunit (GCLC), and glutathione *S*-transferase P 1 (GSTP1) [9]. In healthy adults, consumption of fruits and vegetables (>660 g/day) lowers plasma concentrations of homocysteine and C-reactive protein (CRP) with reduced *NFκB1*, *TNFα*, *IL6*, *IL1R1* and *ICAM1* gene expression, independent of age, gender, physical activity, energy intake, systolic blood pressure, circulating non-esterified fatty acids, body mass index, or smoking [10].

Since fruits and vegetables are comprised of innumerable phytochemicals, reductionist theory has focused on component analysis and the association of biological activity with specific active principles. There are many examples, such as polyphenols, anthocyanins, carotenoids, and lycopene [11]. While work with such phytochemicals is of significant value, it is unlikely that a sufficient quantity of such active principles would actually be provided through the diet. Additionally, the quantity of active principles in a particular fruit or vegetable can vary greatly [12], so it would be difficult to control the amount delivered by dietary consumption. This provides a rationale for the dietary supplement industry as a supplier of pure natural products found in specific fruits or vegetables, as well as clinical trials where high-risk groups are treated with high doses of pure natural products. For example, human trials conducted with resveratrol, a component of grapes [13], may involve administration of daily doses up to 5 g [14]. This has little bearing on dietary intake, as this quantity would translate to the daily consumption of around 50 kg of grapes, or 500 L of red wine.

In addition to resveratrol, which has received wide-ranging attention [15], the potential health benefits of many other grape constituents have been studied [16,17]. These bioactive constituents have the potential for modulating the pathophysiology of various conditions such as diabetes, cancer, inflammatory diseases, obesity, and aging [18]. They may function as scavengers for reactive oxygen species (ROS) generated by oxidative stress [19], and downregulate pathways involving inflammatory responses, ribosomal proteins, the electron transport chain and cholesterol biosynthesis [20]. The scientific literature describing studies conducted with the natural components of grapes or grape products is extensive. In our view, since whole grapes are a common constituent of the diet, it is of importance to investigate the accumulative potential of the numerous component parts [21]. In this context, using grape powder as a well-characterized surrogate of the whole grape, which helps to assure the continuity and reproducibility of research, investigations have been performed to evaluate the effects on atherosclerosis, inflammation, cancer, gastrointestinal health, CNS effects, osteoarthritis, urinary bladder function, and vision [22].

Using a mouse model, one key objective of the present study was to investigate the potential of grapes to modulate adverse effects induced by a high-fat western-pattern diet. It should be noted that the ramifications of fat consumption in the human diet is complex [23]. For example, Mediterranean diets, known for their beneficial effects on health, may include a high portion of calories derived from fat (35–40%), but this is derived from plant and vegetable oils as sources of monounsaturated fatty acids [24]. Similarly, dietary omega-3 fatty acids, derived from sources such as fish, flaxseed, canola, and soy oils, may be advantageous. However, the western-pattern high-fat diet, used as a model for chronic disease in the current work, is characterized for being rich in saturated fats, refined carbohydrates and salt. Continuous consumption of a high-fat western-pattern diet correlates with obesity, a complex condition with multifactorial etiology. In particular, chronic conditions such as heart diseases, type 2 diabetes, and stroke, commonly referred to as metabolic syndrome [25], are associated with obesity. A high-fat diet causes oxidative stress, which is associated with inflammatory and metabolic diseases [26] and adversely affects liver metabolic pathways, in part, through the alteration of gene expression. The expression of cytochrome P450 isozymes is negatively affected, as are other entities involved in steroid metabolism, fatty acid metabolism, arachidonic acid metabolism, and the peroxisome proliferator-activated receptor (PPAR) signaling pathway [27]. Grapes can modulate the expression of genes involved with PPARα signaling and therefore improve fatty acid metabolic homeostasis [28]. Other beneficial effects of grapes are associated with reduced cytokine levels, along with the suppression of other inflammatory markers such as nuclear factor kappa B (NFκB) and an increase in PPAR receptor activity [29].

In addition, there is a positive association between consumption of a western-pattern high-fat diet and nonalcoholic fatty liver disease (NAFLD), which may accompany metabolic diseases such as obesity, insulin resistance and type 2 diabetes. NAFLD is typified to include various histopathological features, steatosis, necrosis, fibrosis, hepatocyte ballooning and mixed inflammatory cell infiltration. In principle, antioxidant agents and free radical scavengers can be of value [24]. As exemplified by the amelioration of ischemic/reperfusion induced organ injury through restoring the balance of oxidant-antioxidant status and regulating the release of inflammatory mediators [25], grapes are capable of mediating such an effect. In addition, diet is considered as a first line treatment for NAFLD [26,27]. Accordingly, we explored the potential of grapes to modulate NAFLD in our high-fat mouse model.

Yet another hallmark of administering a western-pattern high-fat diet to mice is the reduction of lifespan by approximately 34% [30]. Given the prevailing consumption of high-fat western-pattern diets, and indications that dietary grape consumption may alter fat-induced etiology, we performed long-term studies with mice reported to model metabolic anomalies associated with human obesity progression. High-fat and standard semi-synthetic diets were supplemented with 5% grape powder, which is a physiologically relevant concentration. In sum, we report the influence on hepatic gene expression, with emphasis on lipid metabolism, as well as effects on longevity and nonalcoholic fatty liver disease (NAFLD).

## 2. Materials and Methods

### 2.1. Animals and Diets

#### 2.1.1. Semi-Synthetic Diets

To assure the consistency and continuity of experimental and clinical research concerning the biological and physiologic potential of grapes, a freeze-dried powder is manufactured under the auspices of the California Table Grape Commission (Fresno, CA, USA). The grape powder, which serves as a surrogate for fresh grapes, is composed of fresh seeded and seedless red, green and black grapes that are ground and freeze-dried to retain their bioactive compounds. For the current studies, standardized freeze-dried grape powder, as prepared and analyzed as described previously [31], was supplied in vacuum-sealed packets and stored at −20 °C to promote the stability of phytochemical components. To further assure quality, the standard product was subjected to microbial analyses and found to be contaminant-free [31].

Based on the composition of grapes, paired isocaloric diets were custom designed and produced by Envigo (Madison, WI, USA) as follows: 4% fat standard diet (STD; TD.160157), STD + 5% (*w*/*w*) standardized grape powder (STD5GP; TD.160158), 42% fat high-fat diet (HFD; TD.160154) and HFD + 5% (*w*/*w*) standardized grape powder (HFD5GP; TD.160155) (Table 1). As described previously, the addition of grape powder in this fashion to these diets does not significantly affect the rate of consumption [32].

#### 2.1.2. Survival Analyses

Two hundred female C57BL/6J mice were obtained from The Jackson Laboratory (Bar Harbor, ME) at four-weeks-of-age and provided with the STD. At 14-weeks-of-age, mice were randomly assigned to two groups (100 per group) and placed on the HFD or the HFD5GP, respectively. The number of mice per group was selected based on a power analysis conducted using a G*Power *a priori* test (effect size calculated, 0.3; alpha error probability, 0.05; degrees of freedom, 1). Mice were housed in temperature and humidity-controlled cages, with a 12 h light-dark cycle. No other animals were housed in the same room. Food and water were provided *ad libitum* throughout their lifetime. A radio-frequency identification (RFID) microchip (Unified Information Devices Inc., Lake Villa, IL, USA) was implanted in each mouse for permanent identification. The body weight of each mouse was measured every other week over the course of the study, and survival was monitored. This study was conducted in accordance with an Institutional Animal Care and Use Committee (IACUC) protocol approved at Long Island University (protocol number 19-07).

#### 2.1.3. Liver Analyses

Forty female C57BL/6J mice were obtained from The Jackson Laboratory (Bar Harbor, ME, USA) at six- weeks-of-age. At 11-weeks-of-age, the mice were randomly divided into four groups (*n* = 10 per each group) and provided with one of four diets described in Table 1 for the next 13 weeks. The mice were housed in temperature and humidity-controlled cages with HEPA filters and were provided with bio-huts and wooden blocks for rodent enrichment. Water and food were provided *ad libitum* with a 12 h light-dark cycle. RFID microchips were implanted to track the body weight of each mouse until the end of the study. This work was conducted in accordance with an IACUC protocol approved at Long Island University (protocol number 19-07).

### 2.2. Liver Collection

With mice staged for liver analyses (Section 2.1.3), at 24-weeks-of-age, after fasting for 12 h (from 10 p.m. to 10 a.m.), euthanasia was performed with 100% CO_2_ gas. The right lobe and distal part of left lobe of the liver was immersed in RNAlater^TM^ Stabilization Solution (Thermo Fisher, Waltham, MA, USA) for RNA-sequencing. A part of the left lobe was stored in 10% Neutral Buffered Formalin (NBF) for Hematoxylin and Eosin (H&E) staining. The remaining part of the liver, containing the right and caudate lobe, was snap frozen in liquid nitrogen.

### 2.3. Histopathological Examination of Liver

Livers fixed in 10% NBF were transferred to 70% EtOH. Samples were dehydrated in ascending grades of alcohol (70, 95%, and finally absolute alcohol), cleared in xylene, and infiltrated with paraffin at 60 °C using the Leica ASP300S processor. The tissues were then embedded in paraffin molds with the Leica EG1150C. For histopathological evaluation, serial sections were cut at 4 µm thickness and deparaffinized in xylene and graded alcohols (100 and 90%), then stained with H&E (Poly Scientific R&D Corp: S216 and A176), dehydrated in graded alcohols (90 and 100%), cleared in xylene using an automatic Leica autostainer XL, and cover slipped with cytoseal 60 (Thermo Scientific: 8310-4) mounting media.

### 2.4. RNA-Sequencing

Total RNA was isolated from the liver of the four diet groups (Section 2.1.3) using an RNeasy kit (Qiagen, Hilden, Germany), and the samples were shipped to Novogene Corporation Inc. (Sacramento, CA, USA) for RNA-Sequencing. In total, 40 samples were analyzed, 10 from each group (*n* = 10).

#### 2.4.1. Differential Expression Analyses

Differential expression analyses among the four diet groups were performed using the DESeq2 R package (1.14.1) [33]. Adjusted *p* value (Padj), also referred to as false discovery rate (FDR), of 0.05, and |log_2_(fold-change)| of 1, were set as the threshold for significant differential expression.

#### 2.4.2. Gene Ontology Analyses

Gene Ontology (GO) enrichment analyses were performed by comparing the query gene sets of differentially expressed genes (DEGs) among the four diet groups with GO terms curated in the gene ontology resource (http://geneontology.org (accessed on 1 March 2020)), as “biological process”, “molecular function”, and “cellular component” aspects, with Padj < 0.05 considered as significant enrichment.

#### 2.4.3. KEGG and Reactome Pathway Analyses

KEGG and Reactome pathway analyses were performed by comparing the query gene sets of differentially expressed genes (DEGs) among the four diet groups with terms curated in the KEGG database (http://www.kegg.jp/ (accessed on 1 March 2020)) or Reactome database (http://www.reactome.org (accessed on 1 March 2020)) with Padj < 0.05 considered as significant enrichment.

### 2.5. RT-qPCR

The purity and the quantity of total RNA extracted from liver were measured using a Biospec-nano spectrophotometer (Shimadzu). A_260_/A_280_ ratios of all samples were ≥2.0. The reaction step for removing gDNA was conducted as follows: a master mix was prepared using iScript DNase and iScript DNase buffer (provided in iScript gDNA clear cDNA synthesis kit, catalogue number 172-5034). This mixture was added to the RNA samples and incubated at 25 °C for 5 min and 75 °C for 5 min in an Eppendorf Mastercycler pro PCR system. Next, iScript reverse transcription supermix was added to DNase-treated RNA samples and incubated at 25 °C for 5 min, 46 °C for 20 min, and 95 °C for 1 min. The resultant cDNA was used for quantitative reverse transcriptase polymerase chain reaction (RT-qPCR) to measure gene expression levels of *Gstp1* and *Gapdh* (reference gene). iTaq Universal SYBR Green Super mix (Biorad, Catalogue number 1725121), *Gstp1* forward primer (5′-TGGGCATCTGAAGCCTTTTG-3′), *Gstp1* reverse primer (5′-GATCTGGTCACCCACGATGAA-3′), *Gapdh* forward primer (5′-AACGACCCCTTCATTGAC-3′), and *Gapdh* reverse primer (5′-TCCACGACATACTCAGCAC-3′) were used for the procedure. The fluorescence signal from SYBR Green was detected with a LightCycler^®^ 480 Instrument II, and the crossing point (Cp) value of each sample was obtained with LightCycler^®^ 480 analysis software. The relative fold gene expression was calculated using the 2^−∆∆Ct^ method [34].

### 2.6. Statistical Analyses

To determine the statistical significance related to diet and age, two-way ANOVA or two-way repeated measures (RM) ANOVA were performed using SigmaPlot 12.5 (Systat Inc., San Jose, CA, USA). Pearson’s correlation coefficients were computed, and the significance (*r* ≠ 0) was determined by regression analysis using Microsoft^®^ Excel. For comparing regression analyses, the Chow test was conducted using Jupiter notebook 6.0.1, Python3. Values of *p* < 0.05 were considered significant, and detailed values of statistical significance are provided in figure legends and text. For survival analysis, the log-rank test was conducted between the two diet groups using GraphPad Prism 8, considering *p* < 0.05 as significant. The hazard ratio in the survival analysis was computed with the log-rank test approach using GraphPad Prism 8. The hazard ratio for the log-rank test was calculated as (Oa/Ea)/(Ob/Eb), where Oa and Ob represent the number of observed events (deaths) in each group, and Ea and Eb represent number of expected events assuming a null hypothesis of no difference in survival. The standard error of the natural logarithm of the hazard ratio is sqrt(1/Ea + 1/Eb) [35]. Additional statistical methods are described in the text; data are shown as means ± SD.

## 3. Results

### 3.1. Effect of Diets on Mouse Body Weight

**Figure 1:**
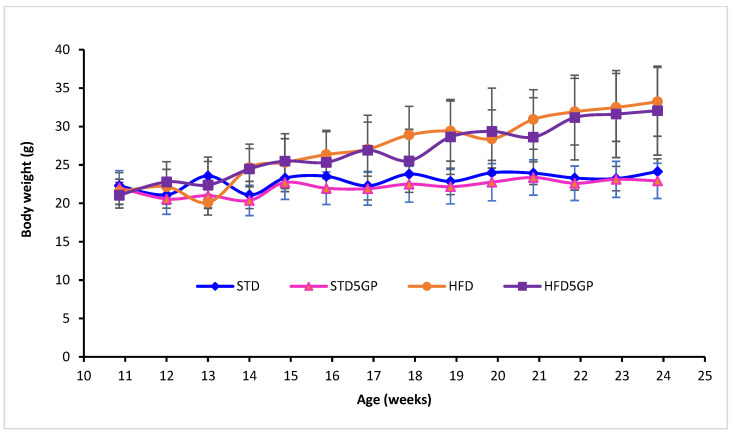
**Dietary fat content but not grape supplementation affects mouse body weight.** Body weights of mice from the beginning of the diet schedule (11 weeks-of-age) for the next 13 weeks (24-weeks-of-age) on standard diet (STD) (*n* = 10), standard diet supplemented with 5% grape powder (STD5GP) (*n* = 10), high-fat diet (HFD) (*n* = 10), or high-fat diet supplemented with 5% grape powder (HFD5GP) (*n* = 10). Prior to 11 weeks of age, all mice were provided with the STD. Mouse body weights were monitored weekly. The body weight of the high-fat diet groups was greater than that of the standard diet groups [two-way ANOVA: F (1, 36) = 53.91, *p* < 0.001]. Within the respective groups, grape supplementation did not significantly alter body weight. Additional details are presented in the text and in Table 2.

As a part of the indirect evaluation of mouse health upon dietary intervention, the body weight of each mouse was recorded every week for 13 weeks (Figure 1). The average body weight of mice (*n* = 40) at the start of diet intervention (week 11) was 21.73 ± 1.82 g, with no significant variation between and within groups following randomization for creating four groups [two-way ANOVA with F (1, 36) = 0.0351 and *p* = 0.852]. Starting at week 17, until the end of the study, mouse body weight increased as a function of age [two-way RM ANOVA; F (13, 468) = 105.971, *p* < 0.001]. At the end of the intervention with respective diets (week 24), the average body weight of mice provided with the HFD (33.23 ± 4.48 g) was significantly greater than the average body weight of mice provided with STD (24.13 ± 1.67 g) [two-way ANOVA with Holm–Sidak multiple comparison *post hoc* test with F (1, 36) = 53.91 and *p* < 0.001] (Table 2). The average body weight of the STD group (24.13 ± 1.67 g) did not differ from the average body weight of the STD5GP group (22.93 ± 2.30 g) (*p* = 0.635, 2-way RM ANOVA), and the average body weight of the HFD group (33.23 ± 4.48 g) did not differ from the average body weight of the HFD5GP group (32.07 ± 5.79 g) (*p* = 0.414, 2-way RM ANOVA). Thus, given that there was no difference found within the standard diet groups (STD and STD5GP) or the high-fat diet groups (HFD and HFD5GP), we conclude that grape powder supplementation of the diets does not have an effect on mouse body weight.

### 3.2. Grape Powder Supplementation of High-Fat Diet Enhances Mouse Longevity

**Figure 2:**
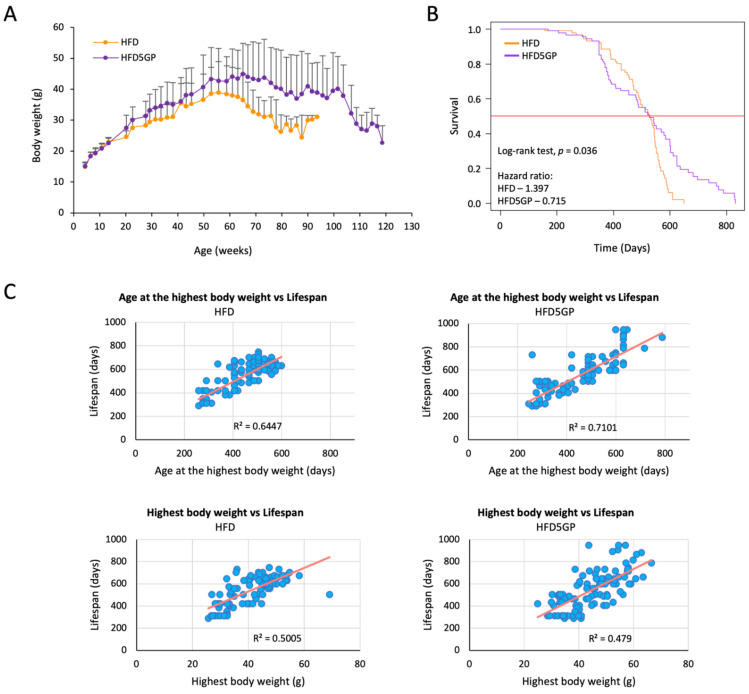
**Grape powder supplementation of a high-fat diet affects temporal weight loss and increases the lifespan of C57BL/6J mice.** (**A**) At 14-weeks-of-age, mice were divided into two groups (*n* = 100 per group), and diet was changed from the STD to either the high-fat diet (HFD) or the high-fat diet supplemented with 5% grape powder (HFD5GP) for the remainder of their lifespan. Body weight was recorded every two weeks and recorded as mean ± SD. (**B**) Kaplan-Meier plot showing the survival of mice provided with the HFD or the HFD5GP. Survival was enhanced with the group provided with the HFD5GP (*p* = 0.036; log-rank test). The hazard ratio of the HFD group was 1.397 (95% CI, 0.99–1.96), whereas the hazard ratio for the HFD5GP group was 0.715 (95% CI, 0.507–1.009). (**C**) Correlation plots showing the age at highest body weight versus lifespan for individual mice (**upper** panels), and the highest body weight attained by individual mice versus lifespan (**lower** panels).

In order to assess the effect of supplementing a high-fat diet with grape powder, a lifelong dietary study was performed with C57BL/6J mice (Section 2.1.2). Throughout the course of the work, body weight and survival were recorded. As shown in Figure 2A, the body weight of the two groups did not show any difference until about four months-of-age, when mice provided with the HFD5GP began a path of divergence (HFD vs. HFD5GP; *p* = 0.001; Student’s *t*-test). The highest body weight observed for the mice provided with the HFD5GP was 44.86 ± 9.96 g, which is greater than the highest body weight recorded for mice provided with the HFD, 38.90 ± 10.02 g (*p* = 0.002, Student’s *t*-test).

This trend continued until about 100-weeks-of-age, when a diminution of body weight, similar to that observed with mice on the HFD at around 70-weeks-of-age, came into play. This extended maintenance of body weight correlated with survival, as illustrated by the Kaplan-Meier curve presented in Figure 2B. The survival of mice provided with the HFD5GP was significantly enhanced compared to mice provided with the HFD (*p* = 0.036; log-rank test). The hazard ratio of the HFD group was calculated as 1.397 (95% CI, 0.99–1.96), whereas the hazard ratio of the HFD5GP group was 0.715 (95% CI, 0.507–1.009), indicating an increased risk of mortality for mice provided with the HFD.

Furthermore, the correlation analysis of individual mouse body weight maxima as a function of longevity is shown in Figure 2C. For mice provided with the HFD, there was a positive correlation between the age of highest body weight vs. lifespan (*r*^2^ = 0.64, *p* < 0.0001, regression analysis) and overall highest body weight vs. lifespan (*r*^2^ = 0.50, *p* < 0.0001, regression analysis). Similarly, for HFD5GP, there was a significant positive correlation between the age of highest body weight vs. lifespan (*r*^2^ = 0.71, *p* < 0.0001, regression analysis) and the overall highest body weight vs. lifespan (*r*^2^ = 0.47, *p* < 0.0001, regression analysis). Comparing the two groups shown in these regression analyses, there was no significant difference between the overall highest body weight obtained by the HFD group and the HFD5GP groups (*p* = 0.991, Chow test). However, as suggested by visual inspection of Figure 2A, the age at which the highest body weight was achieved appears to be later in life with the HFD5GP group as compared to the HFD group. This same tendency can be gleaned by inspection of the data shown in Figure 2C, with greater density observed in the upper right-hand quadrants of the HFD5GP groups. Statistical significance was not achieved when comparing age at the highest body weight with lifespan with the HFD5GP and HFD groups (*p* = 0.141, Chow test), but the trend is apparent.

Taken together, these data demonstrate that grape powder supplementation of a HFD significantly improves mouse survival relative to a HFD without grape supplementation, and the cachexia associated with morbidity and mortality is delayed.

### 3.3. Grape Powder Supplementation of High-Fat Diet Reduces Lipid Accumulation in Mouse Liver

**Figure 3:**
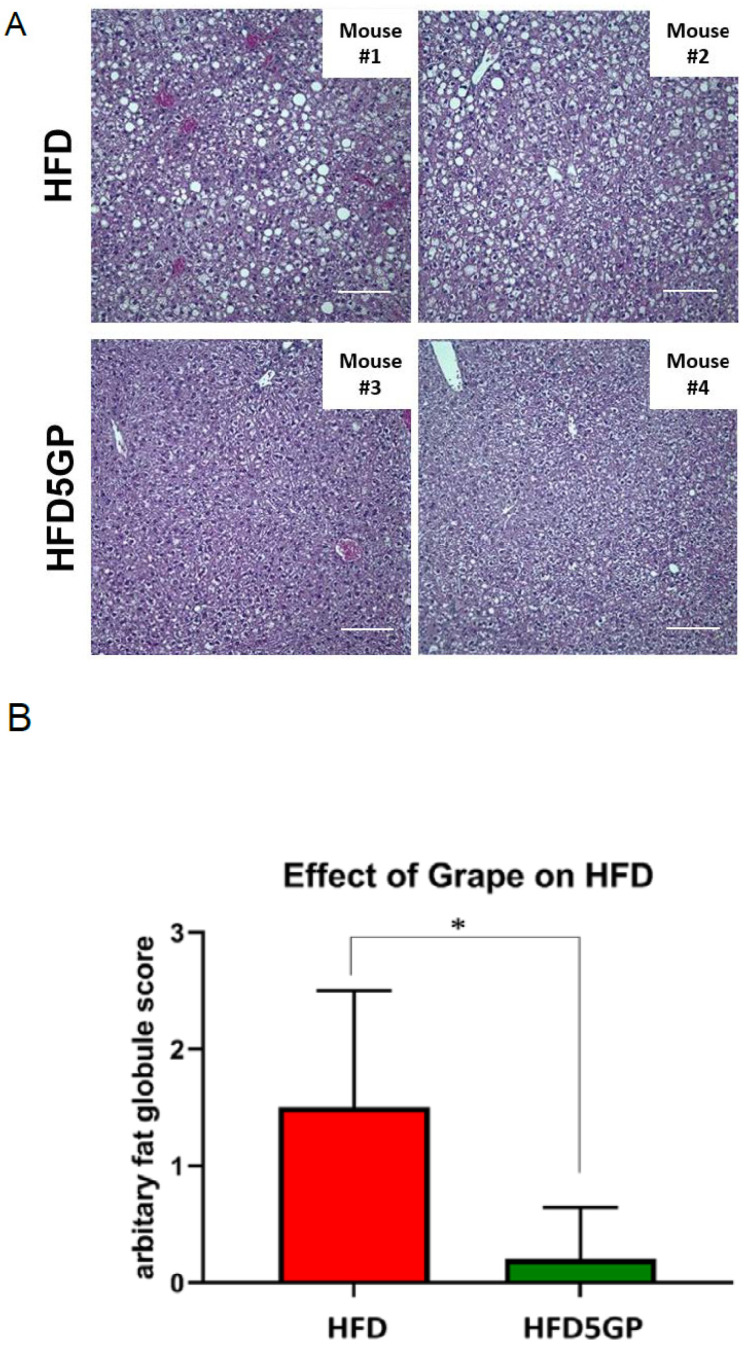
**Effect of grape on hepatic steatosis.** (**A**) Representative H&E images of liver from mice provided with the HFD (1 and 2) and the HFD5GP (3 and 4) (image magnification, 20X). (**B**) Quantitation of the number of fat vacuoles in the liver from mice provided with the HFD (*n* = 5) or the HFD5GP (*n* = 5). Values are presented as mean ± SD. * *p* = 0.032 and U = 2.0 (Mann-Whitney U test).

As a high-fat content of diet is a known cause of lipid accumulation leading to hepatic steatosis, we were interested in examining the potential effect of supplementing the HFD with grape powder. Toward this end, mice that had been provided with the HFD or with the HFD5GP for 13 weeks were fasted for a period of 12 h [36] and euthanized. Liver was harvested and processed for histological examination by H&E staining. Representative images of H&E-stained samples are shown in Figure 3A. By visual inspection, fat vacuoles indicative of lipid accumulation was clearly apparent in the specimens obtained from the HFD group. For quantification, as described previously [37], fat vacuoles observed in the samples were scored as follows: −,0; +/−,0.5; +,1; ++,2; +++,3. As illustrated in Figure 3B, liver samples from mice provided with the HFD had a significantly greater number of fat vacuoles compared to those from mice provided with the HFD5GP. These data suggest that the addition of grape powder to the HFD has the potential of reducing or preventing the extent of hepatic steatosis.

### 3.4. Dietary Fat Content and Supplementation with Grape Powder Alters Gene Expression Profiles in Mouse Liver

**Figure 4:**
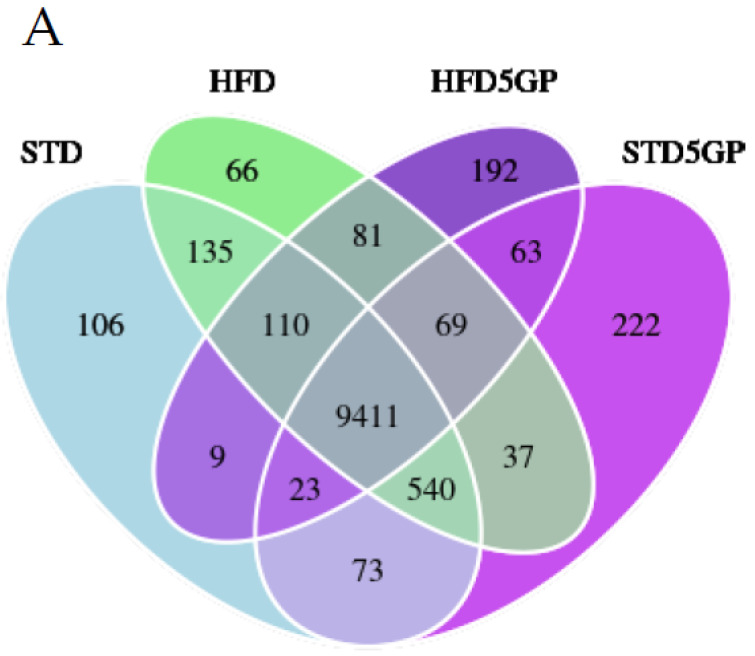

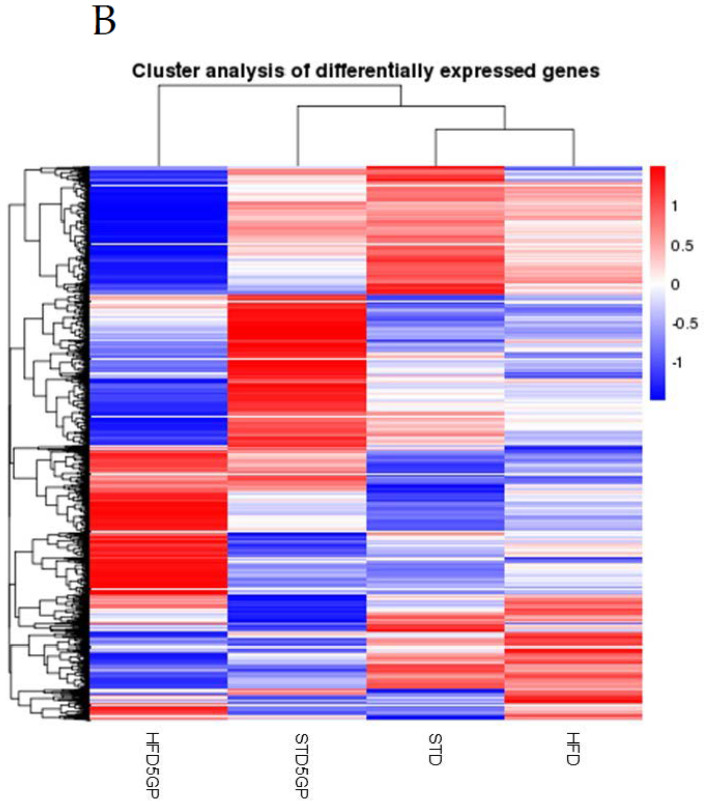
**Dietary fat content and grape powder supplementation alters gene expression profiles in mouse liver.** (**A**) Venn diagram of genes expressed by the four groups of mice illustrating genes co-expressed (overlapping regions) and uniquely expressed (non-overlapping regions) among all groups. The default threshold of FPKM value is set to 1 for the selection of the genes for each group. (**B**) Heat map and cluster analysis showing unique gene expression profiles for each of the four groups of mice. Clustering analysis was carried out by Novogene Corporation Inc. (Sacramento, CA, USA) using a build-in R package, heatmap. Focus is placed on data (Union_for_cluster.xls) which is the union gene set of all comparison groups. Relative gene expression levels and −log2(ratios) are used for clustering. The clustering calculates the distance between each gene and evaluates the relative gene distance through iteration. Finally, genes are divided into several subgroups according to gene distance. H-cluster, K-means and SOM are the main clustering methods used, implemented in R language (Version 1.0.12).

To determine the effect of dietary fat content and grape powder supplementation on gene expression, we performed RNA-Seq analysis with RNA isolated from liver derived from each of the four groups of mice described in Section 2.1.3 (*n* = 40). The Venn diagram shown in Figure 4A illustrates the number of genes (FPKM > 1) that are uniquely expressed within each group as well as overlapping regions that show the number of genes co-expressed in two or more groups. Interestingly, the HFD and HFD5GP groups demonstrated the unique expression of 66 and 192 genes, respectively, whereas the STD and STD5GP groups demonstrated the unique expression of 106 and 222 genes, respectively. To further compare the gene expression patterns of the four diet groups, a differentially expressed gene (DEG) list was used for cluster analysis and the generation of a heatmap. Each group demonstrated a distinctive gene expression profile, as clearly shown in Figure 4B. Remarkably, a cluster analysis revealed the gene expression profile of the STD group maps in a relatively similar fashion to the HFD group. Adding grape to the STD alters the expression pattern, with cluster analysis placing the STD5GP group adjacent to the STD and HFD5GP groups. The most profound difference was observed with the HFD5GP group, which is substantially different from that of HFD and STD5GP groups, but mapped somewhat closer to the STD5GP group, as suggested by cluster analysis (Figure 4B). These data demonstrate that a change in diet composition, whether in terms of fat content or grape powder supplementation, alters gene expression profiles in the liver.

### 3.5. High Dietary Fat Content Alters Gene Expression in Mouse Liver

**Figure 5:**
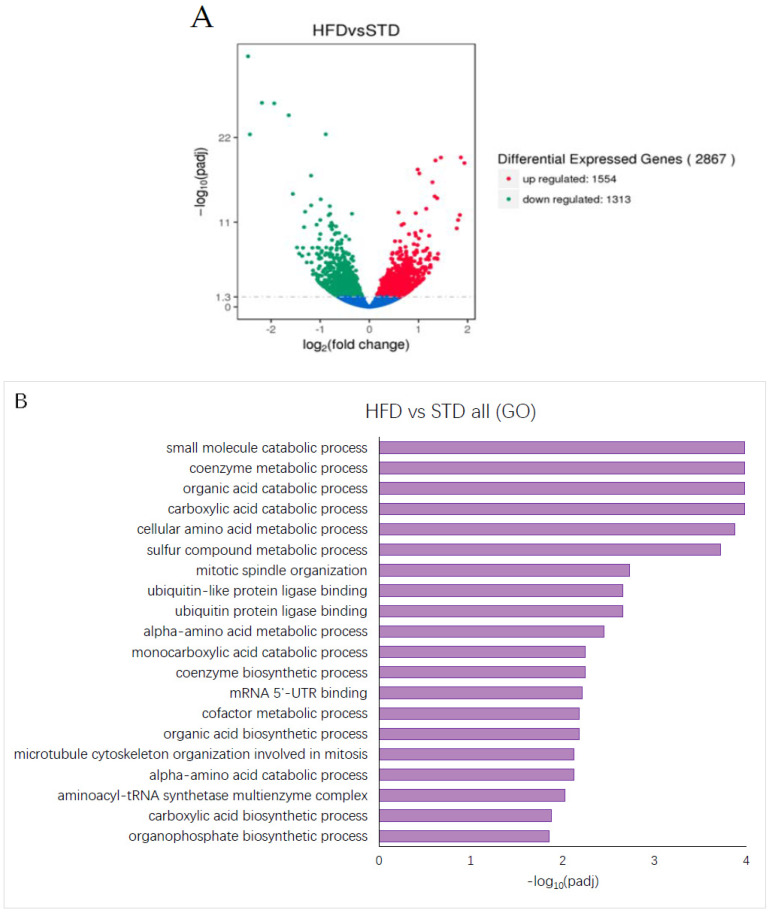
**HFD alters gene expression in mouse liver.** (**A**) Volcano plot showing upregulated (red dots) (1554), downregulated (green dots) (1313) and unaltered (blue dots) genes in liver derived from the HFD group compared to the STD group. The threshold for differentially expressed genes was set as |log_2_(fold-change)| > 1 and −log_10_(Padj) > 1.3 (Padj < 0.05). The plot illustrates the upregulation of 1554 genes and the downregulation of 1313 genes. (**B**) GO analysis showing significantly enriched terms for the gene set differentially expressed in the liver of mice from the HFD group compared to the STD group. −log_10_(Padj) > 1.3 (Padj < 0.05) was considered as significant enrichment.

Changing the diet composition to a high-fat content leads to altered metabolic processes in the liver. Comparison of RNA-Seq data derived from the HFD and STD groups shows that a change in fat content in the diet leads to the upregulation of 1554 genes and the downregulation of 1313 genes, yielding a total of 2867 DEGs (Figure 5A). Using these 2867 DEGs for GO enrichment analysis, in the liver of mice provided with the HFD, a variety of metabolic processes appear as significantly enriched (Figure 5B). Pathway analysis using the KEGG and Reactome databases did not reveal any statistically significant enrichment of processes or pathways in the liver of mice fed a diet with high-fat content (data not shown).

### 3.6. Grape Powder Supplementation Alters Gene Expression in Mouse Liver

**Figure 6:**
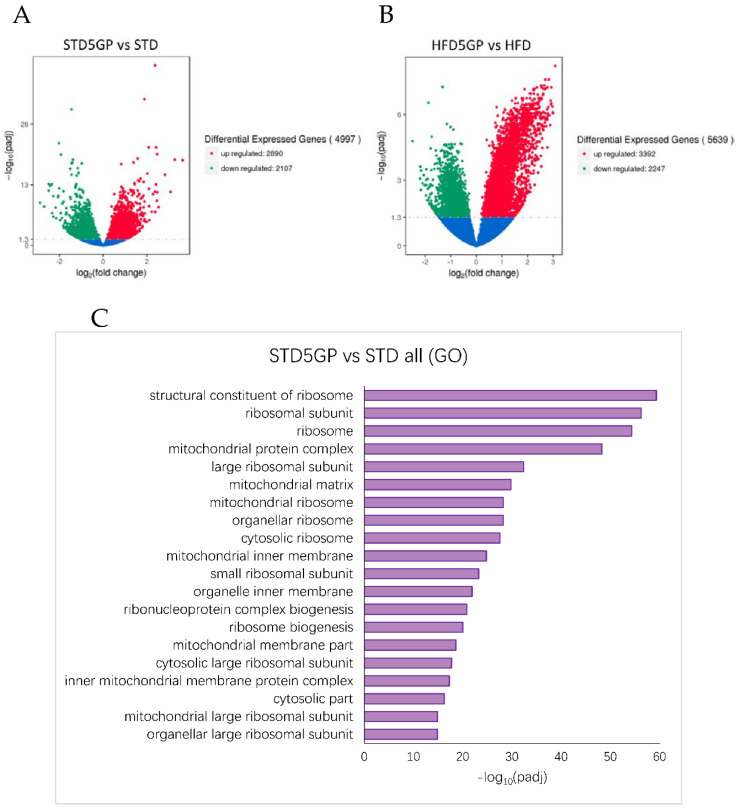

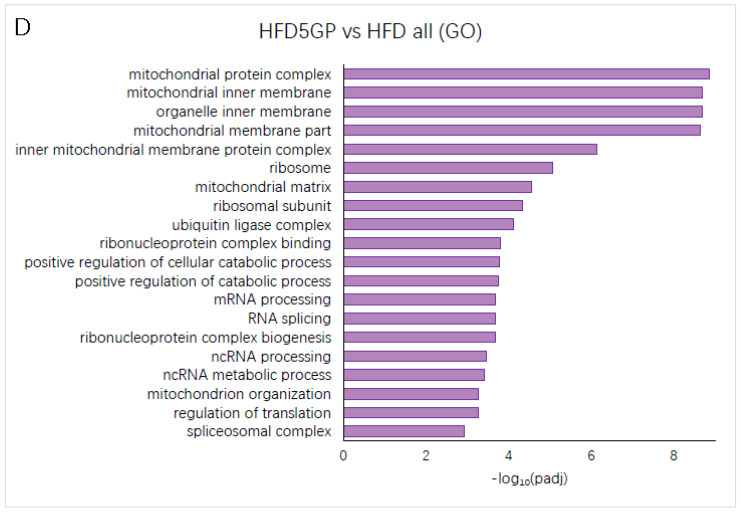
**Grape powder supplementation of diet alters gene expression in mouse liver irrespective of fat content.** (**A**) Volcano plot showing upregulated (red dots) (2890), downregulated (green dots) (2107), and unaltered (blue dots) genes in the liver of mice provided with STD5GP compared with that of mice provided with STD. The threshold for differentially expressed genes was set as |log_2_(fold-change)| > 1 and −log_10_(Padj) > 1.3 (Padj < 0.05). (**B**) Volcano plot showing upregulated (red dots) (3392), downregulated (green dots) (2247), and unaltered (blue dots) genes in the liver of mice provided with HFD5GP compared with that of mice provided with HFD. The threshold for differentially expressed genes was set as |log_2_(fold-change)| > 1 and −log_10_(Padj) > 1.3 (Padj < 0.05). (**C**) GO enrichment analysis showing the top significantly enriched terms for the gene set differentially expressed in the liver of the STD5GPgroup compared with the STD group. (**D**) GO enrichment analysis showing the top significantly enriched terms for the gene set differentially expressed in the liver of the HFD5GP group compared to the HFD group. −log_10_(Padj) > 1.3 (Padj < 0.05) was considered as statistically significant enrichment.

**Figure 7:**
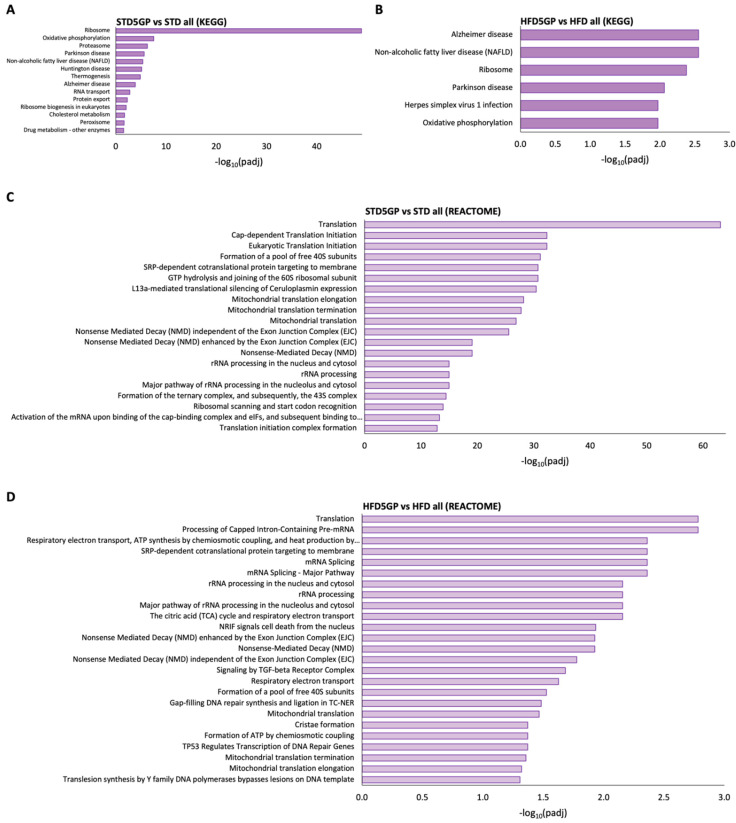
**Dietary grape powder supplementation shows enrichment for terms/pathways associated with translation in mouse liver irrespective of fat content.** (**A**) KEGG pathway analysis showing the top significantly enriched terms for the gene set differentially expressed in the liver of the STD5GP group compared with the STD group. (**B**) KEGG pathway analysis showing the top significantly enriched terms for the gene set differentially expressed in the liver of the HFD5GP group compared with the HFD group. −log_10_(Padj) > 1.3 (Padj < 0.05) was considered as statistically significant enrichment. (**C**) Reactome pathway analysis showing the top significantly enriched terms for the gene set differentially expressed in the liver of the STD5GP group compared with the STD group. (**D**) Reactome pathway analysis showing the top significantly enriched terms for the gene set differentially expressed in the liver of the HFD5GP group compared with the HFD group. −log_10_(Padj) > 1.3 (Padj < 0.05) was considered as statistically significant enrichment.

RNA-Seq data obtained from the liver of grape powder supplemented dietary groups were compared to non-supplemented dietary groups. Relative to the STD group, the STD5GP group demonstrated the upregulation of 2890 genes and the downregulation of 2107 genes, yielding a total of 4997 DEGs (Figure 6A). Relative to the HFD group, the HFD5GP group demonstrated the upregulation of 3392 genes and the downregulation of 2247 genes, yielding a total of 5639 DEGs (Figure 6B). Using these DEGs as a query gene set for GO enrichment analysis, ribosome, ribosomal subunit, mitochondrial protein complex, mitochondrial inner membrane and several other ribosome and mitochondria associated terms were significantly enriched in the liver of mice provided with the grape supplemented diet (Figure 6C,D). Pathway analysis using the KEGG and Reactome databases also showed ribosome, translation and ribosome and translation associated terms as significantly enriched in the liver of mice provided with the grape supplemented diet (Figure 7A–D). These data demonstrate that supplementation of the diet with grape powder, irrespective of fat content, leads to altered gene expression in the liver.

### 3.7. Grape Powder Supplementation of High Fat Diet Alters Genes Responsible for Lipid Metabolism in Mouse Liver

To explore the molecular mechanism related to the effect of grape powder supplementation in reducing HFD-induced lipid accumulation in mouse liver, we mined our RNA-Seq data of liver from the HFD and HFD5GP groups for genes involved in lipid metabolism. It was found that genes involved in the transportation of free fatty acids (FFA) to the site of degradation, such as *Fabp1* [38], and in mitochondrial degradation of FFA, such as *Acads*, *Atp5j*, *Atp5j2*, *Atp5k*, and *Atp5l* [39,40,41], were significantly upregulated in the liver of the HFD5GP group compared to the HFD group (Table 3). In addition, we found that the genes such as *Mogat1* [42], involved in phospholipid esterification [43], were also significantly upregulated in the liver of mice provided with the HFD5GP compared to that of mice provided with the HFD (Table 3). Furthermore, we found that the genes involved in sequestration of FFA, such as *Plin3* and *Plin5* [44], in hydrolysis of FFA remaining after sequestration [45], such as *Abhd16a*, and *Abhd17b* [46], were also significantly upregulated in the liver of mice provided with the HFD5GP compared to those of mice provided with the HFD (Table 3). On the other hand, we found that genes such as *Plin4, Acaa1b* [47], and *Slc27a1*, associated with lipid content [48], cholesterol synthesis and redistribution of lipids from fat and muscle to liver [49], were significantly downregulated in the liver of the HFD5GP group compared with that of the HFD group (Table 3). Taken together, these data demonstrate that grape powder supplementation of the HFD modulates the expression of genes involved in lipid metabolism and thereby may lead to reduction in HFD-induced lipid accumulation in mouse liver.

### 3.8. Grape Powder Supplementation of Standard but Not High-Fat Diet Enhances Antioxidant Potential in Mouse Liver

**Figure 8:**
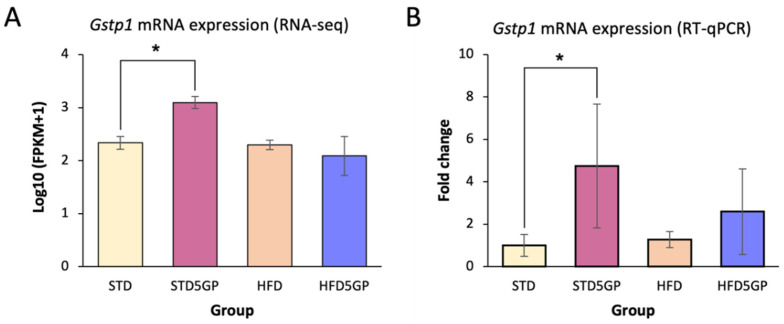
**Grape powder supplementation enhances *Gstp1* expression in mouse liver.** (**A**) Log_10_ (FPKM+1) values for *Gstp1* expression derived from RNA-Seq data obtained with the liver of mice from the STD5GP group compared with liver of mice from the STD group (* *p* < 0.0001; two-sample two-tailed *t*-test). Comparison of the HFD and HFD5GP groups revealed no difference (Padj = 0.782). (**B**) RT-qPCR analysis of *Gstp1* expression in the liver of mice from the STD5GP group compared to liver of mice from the STD group (* *p* = 0.001; two-sample two-tailed *t*-test), and from liver of mice from the HFD5GP group compared with liver of mice from the HFD group (*p* = 0.068). Data are presented as mean ± SD of three independent experiments.

A large number of phytochemicals are known to exhibit antioxidant activity. To provide a preliminary assessment of the potential of dietary grape supplementation to mediate such a response, we mined our RNA-Seq data of liver from the STD, STD5GP, HFD, and HFD5GP groups and examined the expression of *Gstp1*, a known regulator of oxidative stress [50]. It was found that *Gstp1* was significantly upregulated in the liver of mice provided with the STD5GP compared to mice provided with the STD (Figure 8A). Based on RNA-Seq data, the difference in the expression of *Gstp1* when comparing the HFD5GP and HFD groups was not statistically significant (data not shown). We further examined *Gstp1* expression by performing RT-qPCR analysis with RNA isolated from liver of the four dietary groups. *Gstp1* expression was significantly increased, by about five-fold, in the liver of mice of the STD5GP group compared with that of the STD group (Figure 8B). Enhancement of expression was evidenced with liver from the HFD5GP group compared with that of the HFD group, but statistical significance was not quite achieved with these experimental conditions (*p* = 0.068).

In that these data demonstrate the potential of grape powder supplementation to enhance *Gstp1* expression, we examined some additional genes operating under the control of the antioxidant-response element (ARE) by mining our RNA-Seq data. Relative to the STD and HFD groups, the grape diet enhanced the expression of glutathione peroxidase 4 (*Gpx4*) in the STD5GP [log_2_(1.285); Padj, 0.00004] and HFD5GP [log_2_(2.401); Padj < 0.0001] groups. In addition, relative to these controls, grape diet elevated the expression of glutathione synthetase (*Gss*) in the STD5GP [log_2_(1.886); Padj < 0.0001] and the HFD5GP [log_2_(0.812); Padj, 0.008] groups. Relative to the STD group, glutathione peroxidase 7 (*Gpx7*) [log_2_(0.818); Padj, 0.012] and superoxide dismutase type 1(*Sod1*) [log_2_(0.573); Padj, 0.039] were upregulated in the STD5GP group, and relative to the HFD group, glutathione peroxidase 8 (*Gpx8*) [log_2_(1.189); Padj, 0.003] was upregulated in the HFD5GP group.

## 4. Discussion

Many scientific investigations conducted with whole foods, compounds derived from whole foods, drugs, or other test substances are conducted with in vitro or animal models of acute disease. Controlled chronic consumption of a high-fat diet also represents a model of disease, but the ramifications are less acute. For this work, we elected to employ female C57BL6/J mice. These mice are known to be responsive to a high-fat diet [51], and the progression of obesity and metabolic anomalies resembles the condition of human obesity [52]. The administration of a high-fat diet to these mice reduces their lifespan by approximately 34% [30].

The main focus of our work was to investigate the potential of dietary grapes, as a whole food, to modulate some parameters associated with high-fat diet consumption. For this purpose, we utilized a standardized whole grape product that is representative of what is found in the human diet rather than studying a single phytochemical known to be in grapes, such as resveratrol. To limit experimental variables, we used a semi-synthetic standard diet as a base, which is devoid of confounding constituents that are present in commercial animal chow. Furthermore, we elected to supplement the high-fat semi-synthetic diet with 5% grape powder. Although translations of dose between species is not an exact science, based on body weight, daily consumption rates, and metabolic correction factors [53], it was estimated that supplementation of the mouse diet with 5% grape powder corresponds to the daily consumption of about 300 g of fresh grapes by a human being weighing 70 kg. This quantity corresponds to roughly 2.5-times a normal ¾ cup serving of grapes, each of which is approximately 125 g.

One part of our study was designed to investigate the effect of adding grape powder to a standard diet. Remarkably, based on RNA-Seq analysis, unique genetic expression profiles were observed with each dietary group. Relative to the STD5GP group, 109 unique genes were expressed in the STD group, and relative to the STD group, 222 unique genes were expressed in the STD5GP group. Cluster analysis revealed distinct differences between the two groups, which was accentuated by the differential expression of 4997 genes.

Results generated by data mining tools, including GO, KEGG and Reactome, suggest that dietary grape supplementation (STD5GP) significantly increased structural integrity in the ribosome, mitochondrial protein complex, organelle inner membrane, and protein translation-related pathways when compared to the control group (STD). The enrichment of ribosome related genes, which are primarily involved in the translation of genetic information [54] and cell survival [55]. Enriched ribosomal genes are associated with processes such as cell growth, differentiation, proliferation and biological development [54]. Dysregulation from these processes can potentiate diseases such as Diamond–Blackfan anemia, Schwachman–Diamond syndrome, and cancer, including hepatocellular carcinoma [56].

Although not listed in the figures as top-ranked pathways, drug metabolism, glutathione, detoxification, and oxidative stress-associated pathways were significantly enriched in the STD5GP group, as follows: response to oxidative stress (GO:0006979) (Padj < 0.0039), glutathione metabolic process (GO:0006749) (Padj < 0.006), glutathione transferase activity (GO:0004364) (Padj < 0.025), drug metabolism—other enzymes (KEGG: mmu00983) (Padj < 0.029), cellular responses to stress (R-MMU-2262752) (Padj < 0.00001), and detoxification of reactive oxygen species (R-MMU-3299685) (Padj < 0.0045).

Since *Gstp1* is listed as a common component of these pathways, we investigated this species in further detail. Grape supplementation significantly elevated the expression of *Gstp1* in the STD5GP group compared with the STD group, suggesting a greater potential for metabolic detoxification. This is of interest since GSTs have an established role in metabolizing xenobiotics and protection against procardiogenic factors, along with the detoxification of ROS. This, in turn, let us to investigate some related species of relevance through the mining of our RNA-Seq data. Relative to the STD and HFD groups, the grape diet enhanced the expression of glutathione peroxidase 4 (*Gpx4*), glutathione synthetase (*Gss*), glutathione peroxidase 7 (*Gpx7*), superoxide dismutase type 1 (*Sod1*), and glutathione peroxidase 8 (*Gpx8*). Oxidative stress can be regarded as a primary factor leading to pathological conditions such as diabetes, neurodegenerative diseases, and cardiovascular diseases [57], and the species listed here potentiate the maintenance of a redox cellular state [58].

Of course, a multitude of adverse consequences is associated with the chronic consumption of a high-fat diet. Of major concern is the initiation of nonalcoholic fatty liver disease (NAFLD). Our data suggest that grape has the potential to abate nonalcoholic fatty liver (NAFL), which can lead to liver damage/enlargement or other liver complications. NAFLD is estimated to affect about 25 percent of adults in the world. In our 24-week study, the average body weight of mice provided with the HFD5GP (32.07 ± 5.79 g) did not differ from those provided with the HFD (33.23 ± 4.48 g). However, gross histological examination of the liver revealed the diminution of a hallmark associated with NAFL, vacuoles indicative of lipid accumulation. This important observation requires additional investigation, but the comparison of genetic expression profiles obtained with liver obtained from the HFD and HFD5GP groups is worth consideration.

First, on a global level, the comparison of the HFD and HFD5GP groups demonstrated the expression of 66 and 192 unique genes, respectively. Of further interest, cluster analysis suggests that variation between the HFD5GP and HFD groups differs to the greatest extent of all the groups tested. As anticipated, there are major differences between the STD and the HFD groups, with 2867 genes differentially expressed, but remarkably, comparison of the HFD group with the HFD5GP group revealed the differential expression of 5639 genes. The implications in terms of pathway modulation can be gleaned from inspection of the data provided herein, some of which suggest the reduction of fatty liver, including mitochondrial and peroxisomal degradation, esterification, phospholipid metabolism, sequestration and hydrolysis.

The introduction of a high-fat diet leads to widespread changes in catabolic processes [59] which, as suggested here by GO analysis, was positively regulated by grape supplementation. GO analysis also indicated grape supplemented HFD led to the enrichment of mitochondrial energy metabolism, which may be related to cell growth, differentiation and development [60].

Given that a high-fat diet is clearly detrimental to health, trending toward a homeostatic state should be of benefit [61], and dietary grape supplementation may be helpful in this regard. Of particular note, the two top categories indicated by KEGG pathway analysis are Alzheimer’s disease and NAFLD. A cadre of genes are known to participate in the generation of NAFLD, and lipid metabolism in general, including those listed in Table 3. Here we show the statistically significant modulation of several relevant genetic entities. Briefly, grape effectively upregulated the expression of FABP1, which is responsible for the transportation of fatty acids to degradation sites [38]. Genes including *Acads*, *Atp5j*, *Atp5j2*, *Atp5k* and *Atp5l* were upregulated by grape supplementation, suggesting the mitochondrial degradation of fatty acids [39,40,41]. The gene responsible for esterification, *Mogat1* [42], was upregulated in the HFD5GP group. Additional genes upregulated in the HFD5GP group are related to FFA sequestration {(*Plin3*, *Plin5*) [44] (*Abhd16a* and *Abhd17b*) [46]}. Furthermore, grape ameliorated adverse gene expression caused by a high-fat diet through the downregulation of *Plin4*, *Acaa1b* and *Slc27a1*, associated with lipid content [48], cholesterol synthesis [47] and redistribution of lipids from fat and muscle to liver [49], respectively. In sum, relative to the HFD group, the gene expression levels observed with the HFD5GP group are consistent with lipolysis, which eventually may lead to the reduction of NAFL.

We have yet to investigate the effects of grapes on gene expression related to Alzheimer’s disease in our mouse model. However, using the same model, we have shown behavior changes and modulation of gene expression in mouse brain as a result of dietary grape administration [62]. Moreover, in a six month clinical trial, Silverman and coworkers reported that the daily administration of 72 g of grape powder (the same powder preparation used in the current study) had a protective effect on brain metabolism [63].

Of additional interest, interrelationships of NAFLD and Alzheimer’s disease have been described in the literature. Many studies have been performed with laboratory animals to investigate this relationship. For example, with a mouse model, Kim et al. demonstrated that chronic NAFLD induced advanced pathological signs of Alzheimer’s disease [64]. More notably, this correlation has been supported by investigations involving human subjects. In a nationwide cohort study involving over four million subjects, is was found that NAFLD is associated with an increased risk of dementia [65]. Furthermore, in a study sample including participants from the offspring and third generation of the Framingham Study, a possible association between liver fibrosis and early Alzheimer’s disease markers was observed [66]. Clearly, it would be of future interest to study the molecular aspects of this interrelationship in greater detail.

In addition to the above, we performed a preliminary study to assess the potential of lifetime dietary grape supplementation to modify longevity with this mouse model. We will note from the outset that performing similar studies with male mice, in addition to female mice, will be of value, and various experimental designs are feasible. This work was performed as a pilot. Long-term studies are currently underway to investigate the effect of gender and the inclusion of grape supplementation later in life, with and without high-fat contained in the diets. Nonetheless, since it is well known that a high-fat diet reduces the lifespan of C57BL6/J mice [30], in the current lifelong study, survival of the group of mice provided with the grape supplemented diet (HFD5GP) was compared with the survival of the HFD group. It was found that the HFD5GP group showed significant improvement relative to the HFD group. The overarching cause of this enhancement remains to be defined, but some of the ancillary results provided herein may be relevant. In particular, alteration of genetic expression and factors related to NAFLD by dietary grapes may help to ameliorate the adversity caused by high-fat consumption.

It is also of some interest to consider body weight as related to longevity. While it is certainly clear that obesity correlates with chronic illness and reduced lifespan, the overall ramifications of body mass and health are complex [67]. It is intriguing that gradual movement from normal body weight in early life to overweight in later life may be associated with decreased mortality risk [68]. Furthermore, obesity itself may be classified as various subtypes: metabolically abnormal obese, metabolically healthy obese and sarcopenic obese [69]. To some extent, these confounding factors may be reflected in our mouse study. In both the HFD and the HFD5GP groups there was a strong correlation between body weight and longevity. Furthermore, the highest body weight achieved by the HFD5GP group appeared to be attained later in life relative to the highest body weight of the HFD group. Of course, correlation does not establish a cause-and-effect relationship, but these are interesting observations that require further in depth attention.

For example, in comparison to control mice, bromodomain containing 2 (*Brd2*)-knockdown mice show severe obesity, but they concurrently display a reduction in obesity-induced inflammatory responses, insulin resistance, glucose intolerance and pancreatic beta cell dysfunction [70,71], as well as extended healthspan and lifespan [72]. In the current study, significantly enriched pathways in the HFD5GP group include modification-dependent protein binding (GO:0140030) and histone binding (GO:0042393), both of which involve *Brd2*, which shows signs of downregulation in the HFD5GP group [log2 (−0.28); Padj, 0.03]. Other genes of interest include adiponectin receptors (*Adipor*) 1 and 2, which are included in the pathway of non-alcoholic fatty liver disease (KEGG: mmu04932), which was enriched with the HFD5GP group. *Adipor1* and *Adipor2* are known to be downregulated in obesity-related insulin resistance. Furthermore, it is reported that the overexpression of either adiponectin receptor isoform in mouse liver is sufficient to improve ceramidase activity, total body glucose metabolism, and hepatic insulin sensitivity, while suppressing hepatic steatosis, in comparison to wild-type control animals [73]. In the current study, the modest enhancement of *Adipor1* [log2 (0.76); Padj 0.001] and *Adipor2* [log2 (0.48); Padj 0.034] was observed with the HFD5GP group, further suggesting a mechanistic unpinning for improving healthspan.

In sum, as a common dietary component, grapes have a high safety profile, allergy is rare, and accumulating evidence indicates some health benefits [21,22,74,75]. The aim of this study was not to evaluate a profound drug-like effect such as a cure for cancer, for example, as described in *The Grape Cure* [76]. Nor was our interest in studying a specific target, such as the inhibition of a particular enzyme or molecular entity. Our goal was to provide a glimmer into what is happening at a holistic level, in the milieu of a living mammal, when chronically administering a relevant quantity of the whole product through the diet.

The results amply demonstrate the profound effect of nutrigenomics, which implicitly suggest the potential for downstream alteration of physiological responses. In fact, we have confirmed alterations in hepatic and urinary metabolite patterns based on gas chromatography-mass spectrometry-based metabolomics. The grape diet was found to reprogram gut microbiota metabolism, attenuate the hepatic oxidative stress of a high-fat diet, and increase the efficiency of glucose utilization by the liver for energy production [77].

In ancillary studies, we have described the effect of grape consumption on mouse brain gene expression and behavior [62]. Currently, we focus on liver, longevity and the western-pattern diet, and report subtle yet intriguing responses. This brings to mind the proverbial saying "You are what you eat", originally attributed to the French lawyer Athelme Brillat-Savarin (*ca.* 1826). It is likely that the concept was inspired by the nutritive value of food, as well as the metabolic conversion of food into human cellular and body parts. The notion of diet leading to phenotypic changes, which in turn may alter our intrinsic character, adds another dimension and even more profound significance to the axiom "You are what you eat". We do not suggest that phenotypic changes are induced solely by grapes, or solely in the liver. We were able to study this due to the availability of suitable tools and materials, and the results are interesting and meaningful. However, these studies lead us to question how much we know about the way in which our routine behavior affects the intricacies of our human form.

## 5. Conclusions

The salient features of the current study include: (1) Standard or high-fat diets yield unique gene expression patterns that each are modulated by the addition of grapes; (2) The addition of grapes to a high-fat diet yields a genetic expression pattern more similar to a standard diet than to a high-fat diet; (3) Dietary grape supplementation reduces histological signs of fatty liver; (4) Genes responsible for the metabolism, transportation, hydrolysis and sequestration of fatty acids are upregulated by the addition of grapes to a high-fat diet; (5) Genes operating under the control of the antioxidant-response element (ARE) are enhanced by the addition of grapes to standard or high-fat diets; and (6) The life-long addition of grapes to a high-fat diet increases longevity. These data illustrate the extraordinary influence of nutrigenomics, a burgeoning field of investigation that will augment our appreciation of diet and health.

## Figures and Tables

**Table 1:** Dietary constituents: 4% fat standard diet (STD; TD.160157); STD + 5% (*w*/*w*) standardized grape powder (STD5GP; TD.160158); 42% fat high-fat diet (HFD; TD.160154); and HFD + 5% (*w*/*w*) standardized grape powder (HFD5GP; TD.160155). Diets were produced by Envigo (Madison, WI, USA).

	Standard Diet (TD.160157) ^3^	Standard Diet with 5% Grape Powder (TD.160158) ^3^	High-Fat Diet (TD.160154) ^4^	High-Fat Diet with 5% Grape Powder (TD.160155) ^4^
**Formula (g/kg)**				
Casein	195	195	195	195
DL-Methionine	3	3	3	3
Sucrose	191.1	191.1	191.1	191.1
Dextrose, anhydrous	66.45	44.3	64.45	44.3
Fructose	66.45	44.3	64.45	44.3
Corn Starch	235.03	232.88	167.43	161.37
Maltodextrin	100	100	0	0
Anhydrous Milkfat ^1^	30	29.85	210	210
Soybean oil	10	10	0	0
Cholesterol	0	0	1.5	1.5
Cellulose	50	50	50	50
Mineral Mix, AIN-76 (170,915)	35	35	35	35
Potassium Citrate, monohydrate	4.03	2.69	4.03	2.69
Calcium Carbonate	4	4	4	4
Vitamin Mix, Teklad (40,060)	10	10	10	10
Ethoxyquin, antioxidant	0.04	0.04	0.04	0.04
Grape powder, freeze-dried ^2^	0	50	0	50

^1^ For each 100 g: Total fat, 99.8 g; Saturated fat, 67 g; Trans fat, 2.6 g; Polyunsaturated fat, 3.9 g; Monounsaturated fat, 26.3 g. ^2^ Grape powder is considered to contain 3.71 kcal/g, 3% fat, 88.6% carbohydrate (as a 1:1 mixture of fructose and glucose), 3.58% protein and 9.73 g/kg K^+^. ^3^ Formulated to 3.6 Kcal/g (Protein, 19.1%; Carbohydrate, 70.5%; Fat, 10.4%). ^4^ Formulated to 4.5 Kcal/g (Protein, 15.3%; Carbohydrate, 42.4%; Fat, 42.3%).

**Table 2:** Summary of the body weights for the indicated groups of mice after 13 weeks on respective diets.

Diet Groups	Average (g)	Median (g)
STD	24.13 ± 1.67 ^a^	23.58
STD5GP	22.93 ± 2.30 ^a^	23.36
HFD	33.23 ± 4.48 ^b^	33.27
HFD5GP	32.07 ± 5.79 ^b^	30.91

Average values are given as mean ± SD. There is no significant difference between the values with the same superscripts (*n* = 10). There is a significant difference between the values designated as ^a^ and ^b^ (*p* < 0.001, two-way ANOVA).

**Table 3:** Mouse hepatic genes involved in lipid metabolism that are modulated by grape powder supplementation of the HFD.

Genes	HFD5GP vs. HFD Log_2_(FC) ^1^	HFD5GP vs. HFD Padj Value	HFD vs. STD Log_2_(FC) ^1^	HFD vs. STD Padj Value	Function
*Fabp1*	1.208	0.0001	0.421	0.006	Transportation of FFA for degradation
*Acads*	1.091	<0.0001	0.239	0.043	Mitochondrial degradation
*Atp5j*	1.320	<0.0001	0.294	0.027	Mitochondrial degradation
*Atp5j2*	1.298	<0.0001	0.311	0.053	Mitochondrial degradation
*Atp5k*	1.223	<0.0001	0.461	0.012	Mitochondrial degradation
*Atp5l*	2.558	<0.0001	0.458	0.030	Mitochondrial degradation
*Mogat1*	1.198	0.013	1.149	<0.0001	Esterification
*Plin5*	1.864	<0.0001	0.065	0.743	Sequestration
*Plin3*	1.026	<0.0001	0.083	0.870	Sequestration
*Abhd16a*	1.880	<0.0001	0.261	0.090	Hydrolysis
*Abhd17b*	1.094	0.0005	0.186	0.419	Hydrolysis
*Plin4*	−0.941	0.010	1.861	<0.0001	Associate preferentially with small lipid droplets
*Acaa1b*	−1.440	0.0001	0.510	0.0001	Cholesterol synthesis
*Slc27a1*	−0.981	0.0003	0.645	0.0002	Redistribution of lipids from fat and muscle to liver

^1^ Fold-change (FC).

## Data Availability

The data presented in this study are available on request from the corresponding author. The data are not publicly available due to privacy.

## Abbreviations

ARE, antioxidant-response element; CRP, C-reactive protein; DEG, Differentially Expressed Gene; FDR, False Discovery Rate; FPKM, Fragments Reads Per Kilobase of exon model per Million mapped reads; GCLC, Glutamate cysteine ligase catalytic subunit; GCLM, Glutamate cysteine ligase regulatory subunit; GO, Gene Ontology; GSTA2, Glutathione *S*-transferase A2; GSTM1, Glutathione *S*-transferase mu 1; GSTP1, Glutathione *S*-transferase P 1; HFD, high-fat diet; HFD5GP, high-fat diet containing 5% grape powder; H&E, Hematoxylin and eosin; KEGG, Kyoto Encyclopedia of Genes and Genomes; NAFL, nonalcoholic fatty liver; NAFLD, nonalcoholic fatty liver disease; NASH, nonalcoholic steatohepatitis; NBF, Neutral Buffered Formalin; NFE2L2, Nuclear Factor Erythroid derivative like 2; NFκB, Nuclear factor kappa B; NQO1, NAD(P)H: quinone oxidoreductase I; Padj, Adjusted *p* value; ROS, Reactive Oxygen Species; SOM, Self-organization mapping; STD, standard diet; STD5GP, standard diet containing 5% grape powder.
